# Electromyographic Evaluations of Masticatory Muscle Activity between Patients with Skeletal Class I and III Relationships

**DOI:** 10.1055/s-0042-1758064

**Published:** 2022-12-13

**Authors:** Pakwan Kulchutisin, Thanaporn Sowithayasakul, Jittima Pumklin, Thosapol Piyapattamin

**Affiliations:** 1 Department of Preventive Dentistry, Faculty of Dentistry, Naresuan University, Phitsanulok, Thailand; 2 Department of Restorative Dentistry, Faculty of Dentistry, Naresuan University, Phitsanulok, Thailand

**Keywords:** electromyography, muscle activity, sagittal discrepancy, skeletal malocclusion

## Abstract

**Objective** The aim of this study was to compare the muscle activity of the masseter muscle (MM) and anterior temporal muscle (TA) of patients with skeletal Class I and III during maximum voluntary clenching (MVC) at the intercuspal position (ICP) and during chewing.

**Materials and Methods** Twenty patients were divided into Steiner's skeletal Class I and III groups. MM and TA activity during each task was measured by using surface electromyography. Averaged MM and TA activity during both tasks, symmetry of each muscle activity, synergy between ipsilateral MMs and TAs, and muscle effort were compared.

**Statistical Analysis** Means and standard deviations of intergroup variables were compared by an independent sample *t* -test for parametric evaluations or by the Mann–Whitney U test for nonparametric evaluations. A probability value of *p* less than 0.05 was considered significant.

**Results** Averaged MM activity and muscle synergy during MVC at the ICP in skeletal Class III patients were lower than that in skeletal Class I patients. Neither symmetry nor muscle effort during both tasks was different.

**Conclusion** Masticatory muscle performance of skeletal Class III patients was inferior to that of skeletal Class I patients.

## Introduction

Skeletal malocclusion is a developmental deformity of the maxilla and/or mandible that can lead to dental deformities, bruxism, trismus, breathing obstruction, and digestion disturbances 1 ; also, if left untreated, it results in poor oral health-related quality of life, difficulties in mastication, and temporomandibular disorder (TMD) in some patients. 2 3 4 Skeletal malocclusion is also a factor affecting masticatory muscle functions 2 5 and can cause changes in masseter and temporal muscle activities at rest, during deglutition, during maximum voluntary clenching (MVC), and when chewing. 4 6

MVC is the stage with the highest muscle contraction and is obtained by biting as hard as possible. A report shows TMD patients with lower electromyography (EMG) signals than subjects without TMD during MVC. 7 The chewing position represents the masticatory muscles' dynamic function and EMG signals are different among various skeletal malocclusions. 2

Muscle balance is the correspondence between bilateral muscle activities and indicates muscle equilibrium. Masticatory muscle imbalance is usually observed in patients with skeletal transversal problems 8 9 and causes TMD in some patients. 10 11

Muscle effort is defined by the percentage of muscle activity during chewing, and the muscle activity during MVC is considered 100%. 2 It is observed to be higher in patients with skeletal malocclusion. 12 13 Since a great deal of muscle effort means that more muscle activity is needed for chewing, patients with skeletal malocclusion are at a great risk of developing TMD. 14 15

Two main types of EMG are intramuscular electromyography (iEMG) and surface electromyography (sEMG). The former using a needle electrode can record single motor unit action potentials with fewer artifacts, but is more invasive, which makes patients uncomfortable and can lead to infection. 16 On the other hand, the latter records the muscle electrical potentials by summating the whole muscle's active motor units, and thus, the muscle activity magnitude and duration can be assessed. 17 sEMG can evaluate muscle functions qualitatively and quantitatively during static and dynamic conditions 18 19 and has efficacy in observing muscle posture hyperactivity, functional hyperactivity, muscle spasm, and muscle imbalance. 10 Since usage of sEMG is limited to the detection of signals from only the muscles located adjacent to skin, masseter muscle (MM) and anterior temporal muscle (TA) are most frequently evaluated. 20 The usage of both EMG types is influenced by some biological factors. They include chronological age, in which an isometric contraction is decreased with increasing age, 21 psychological conditions affecting physiological variations in muscular activity, 22 23 body weight, and skin thickness, in which electrical signal conduction is lower in subjects with thick skin or high subcutaneous fat. 24

Reports on patients with skeletal Class I and III relationships during the rest position have shown that the muscle activity of skeletal Class III patients is significantly higher than that of patients with skeletal Class I, 6 25 but without a significant difference during MVC. 6 Patients with a skeletal Class III relationship have a significantly lower MM balance during MVC 26 and during chewing. 27 Dolichofacial subjects are seen with a higher muscle effort resulting in a lower muscle performance 2 but a lower muscle activity during maximum clenching. 28 However, some reports have shown neither significant difference in the MVC among 60 subjects with different skeletal vertical relationships 29 nor significant association between MM and TA activity during rest and bilateral mastication among subjects with different vertical facial types. 30 Previous studies are controversial because of their various sample groups, ages, and methods. Consequently, comparisons of those observations' results might be impractical.

Hence, this clinical study hypothesized that the averaged muscle activity, symmetry, synergy, and muscle effort of MM and TA were significantly different between patients with skeletal Class I and III relationships during MVC at the intercuspal position (ICP) and during chewing.

## Materials and Methods

This study was approved by Naresuan University (NU) Ethical Committee (IRB No. P10016/63). Postsigning the consent form, new orthodontic patients at NU Dental Hospital between August 2020 and February 2021 were chosen. The inclusion criteria were patients with fully erupted permanent teeth (except third molars) and aged between 18 and 35 years old. Those with a body mass index within the obese range (over 30 kg/m ^2^ ), congenital facial deformity or asymmetry, an ongoing or a history of orthodontic therapy, pain during occlusal contact, a history of TMD, a psychological disorder, or either type of dental substitution were excluded.

Orthodontic clinical examination was conducted, and the overjet, overbite, and Angle's classification data were recorded.

### Analyses of Skeletal Sagittal Relationships

Lateral cephalometric radiographs were obtained by using an X-ray machine (Veraview X800; J. Morita Co., Kyoto, Japan) and traced to classify each patient's skeletal sagittal relationship. The patients were then divided into skeletal Class I (0 degree < The angle indicating the sagittal position between the maxilla and the mandible (ANB) < 4 degrees) and III (ANB < 0 degree) groups. Retracing of five lateral cephalograms randomly chosen from each group was performed by the same examiner with a 1-month interval. The reliability of the data from the first and second measurements were assessed by an intraclass correlation coefficient for cephalometric analyses.

### Muscle Activity Recording Procedures

Prerecording procedures included removing all cosmetic products and grease from the superficial part of MM, TA, and the seventh cervical vertebra area by cleaning the areas with 70% ethanol for 30 seconds and asking the patient to sit and relax in a natural position with his head unsupported and his trunk in an erect position on a chair for 10 min.

After MM and TA had been located by palpation, their activity was recorded by using sEMG (BioEMGIII; BioResearch Associates, Inc., Milwaukee, Wisconsin, United States), in association with a fixed interelectrode distance and ground electrodes. Two bipolar surface electrodes were placed on each patient's side. One of them was in the vertical direction along the TA's anterior margin, while the other was parallel to the MM's superficial part from anterior to the mandibular angle.

**Fig. 1:**
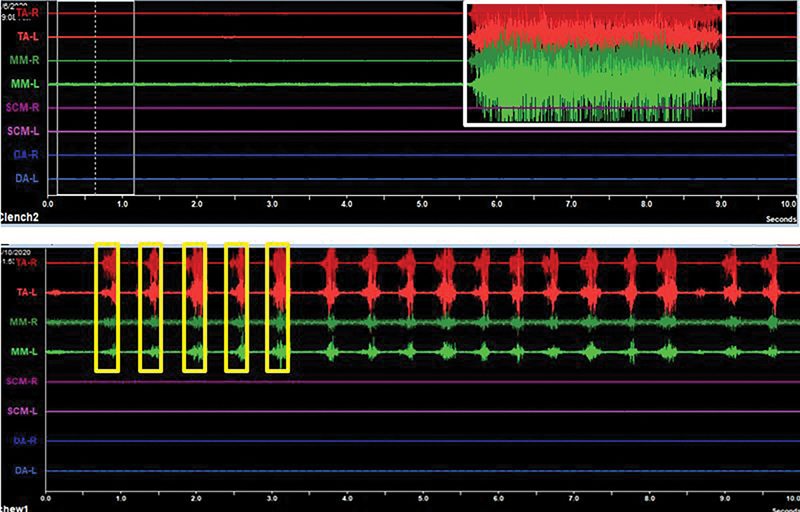
Anterior temporal muscle (TA, red line) and superficial part of masseter muscle (MM, green line) activity of a patient during maximum voluntary clenching at the intercuspal position (upper diagram) recorded at a 3-s interval (white box) and during chewing (lower diagram) recorded at the first five bursts (yellow boxes). Recording time (s) is shown on each figure's abscissa axis. DA, digastric muscle; L, left; R, right; SCM, sternocleidomastoid muscle.

By using the stable electrodes with all cables in fixed positions, sEMG of standard potentials was recorded, followed by those of static and dynamic muscle activity ( Fig. 1 ). After the patient had been asked to clench his teeth as hard as possible for 3 seconds, MVC at the ICP representing a static condition was recorded (white box in the upper diagram of Fig. 1 ). The recording processes were conducted three times with a 3-min resting interval between each session to avoid muscle fatigue. For a dynamic condition, the patient was invited to freely chew a piece of commercially available chewing gum (sized 19 mm × 35 mm; Mondelēz International, Bangkok, Thailand) for 1 min. When the gum had turned soft, the record commenced for 10 seconds. The first five bursts of muscle activity (yellow boxes in the lower diagram of Fig. 1 ) were selected for subsequent analyses.

**Fig. 2:**
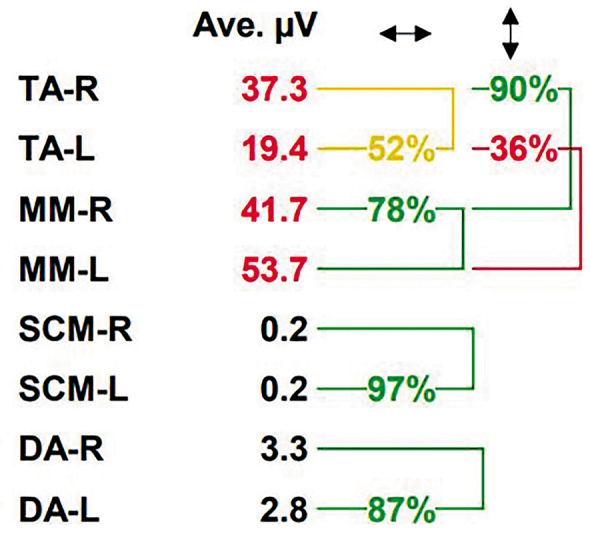
Symmetry (%) of muscle activity of a patient calculated by a BioPAK program. The ↔ column shows the symmetry [left (L) and right (R) balance] of each muscle, while the ↕ column shows the synergy between the superficial part of the masseter muscle (MM) and the anterior temporal muscle (TA). Ave. µV, averaged µV; DA, digastric muscle; SCM, sternocleidomastoid muscle.

Averaged muscle activity (µV) of MM and TA during MVC at the ICP and during chewing, symmetry or left-and-right balance (%) of each muscle activity, and synergy (%) between ipsilateral MM and TA ( Fig. 2 ) were the dependent variables in the study. They were calculated and analyzed by using a BioPAK program (version 8; BioResearch Associates, Inc.) run on a personal computer. In addition, muscle effort (%) was calculated from averaged EMG activity during chewing multiplied by 100 and divided by MVC.

### Statistical Analysis

A Shapiro–Wilk test was used to test the normality of the population data. The obtained data were then analyzed by the Statistical Package for the Social Sciences (SPSS) version 23.0 (IBM; Armonk, New York, United States). Intergroup variables (means and standard deviations) were compared by an independent sample *t* -test for parametric evaluations or by the Mann–Whitney U test for nonparametric evaluations. A probability value of *p* less than 0.05 was considered significant.

## Results

Table 1 shows the characteristics of the 20 patients included in this study ( *n* = 10 for each group). Significant differences in overjet ( *p* = 0.001) and overbite ( *p* = 0.020) were detected between groups but not in their age or body mass index ( *p* > 0.05). A good intraclass correlation coefficient of 0.98 for cephalometric analyses was obtained.

**Table 1:** Characteristics of the patients in each skeletal sagittal group

Characteristics	Skeletal Class I	Skeletal Class III	*p* -Value*
Gender			
Male	3	4	–
Female	7	6	–
Age (years; mean ± SD)	20.97 ± 3.31	20.90 ± 3.19	0.970
Body mass index (kg/m ^2^ ; mean ± SD)	21.18 ± 5.67	23.41 ± 3.90	0.430
Overjet (mm; mean ± SD)	3.65 ± 1.33 ^a^	−1.70 ± 4.22 ^b^	0.001
Overbite (mm; mean ± SD)	2.75 ± 1.40 ^a^	0.80 ± 1.93 ^b^	0.020

Abbreviation: SD, standard deviation.

* Independent sample *t* -test.

Note: Different uppercase letters indicate significant intrarow differences at *p* < 0.05.

Table 2 shows some lower averaged MM and TA activity of the skeletal Class III group than those of the skeletal Class I group during MVC at the ICP. However, only the left and the averaged MM activity between them was significantly different ( *p* = 0.047 and 0.042, respectively). During chewing, the averaged MM and TA activity of the skeletal Class III group was lower than those of the skeletal Class I group, but there was no significant difference ( *p* > 0.05) between them.

**Table 2:** Muscle activity, symmetry of muscle activity, muscle effort, and muscle synergy of the patients in skeletal Class I and III groups (all numerical data are analyzed by an independent *t* -test, except the italicized ones by the Mann–Whitney U test.)

	Skeletal Class I	Skeletal Class III	*p* -Value
**Muscle activity** (µV; mean ± SD)			
Masseter muscle (superficial part)			
During MVC at the intercuspal position			
Left	133.36 ± 77.51 ^a^	73.57 ± 43.20 ^b^	0.047
Right	*126.21 ± 63.80*	*72.98 ± 46.39*	*0.059*
Averaged	129.78 ± 68.53 ^a^	73.27 ± 44.52 ^b^	0.042
During chewing			
Left	*40.16 ± 29.15*	*28.59 ± 22.24*	*0.290*
Right	*40.17 ± 26.43*	*27.86 ± 13.45*	*0.241*
Averaged	*40.16 ± 19.85*	28.22 ± 14.24	*0.070*
Temporal muscle (anterior fibers)			
During MVC at the intercuspal position			
Left	114.98 ± 46.17	72.85 ± 52.32	0.072
Right	120.81 ± 62.38	83.86 ± 41.43	0.136
Overall	117.89 ± 53.26	78.35 ± 46.00	0.093
During chewing			
Left	*36.60 ± 15.30*	*22.66 ± 17.56*	*0.226*
Right	46.12 ± 16.40	37.14 ± 15.77	0.228
Overall	41.36 ± 13.33	31.90 ± 14.17	0.142
**Symmetry of muscle activity** (%; mean ± SD)			
Masseter muscle (superficial part)			
During MVC at the intercuspal position	79.20 ± 11.84	86.20 ± 6.84	0.123
During chewing	54.30 ± 26.42	57.70 ± 20.92	0.245
Temporal muscle (anterior fibers)			
During MVC at the intercuspal position	83.60 ± 15.09	76.80 ± 18.55	0.380
During chewing	*74.30 ± 22.11*	*60.50 ± 24.45*	*0.112*
**Muscle synergy** (%; mean ± SD)			
During MVC at the intercuspal position	* 81.05 ± 9.80 ^a^*	* 72.55 ± 9.13 ^b^*	*0.045*
During chewing	72.20 ± 15.24	72.05 ± 7.31	0.978
**Muscle effort** (%; mean ± SD)			
Masseter muscle (superficial part)	36.11 ± 14.18	44.17 ± 26.26	0.404
Temporal muscle (anterior fibers)	38.86 ± 11.18	48.94 ± 26.01	0.275

Abbreviations: MVC, maximum voluntary clenching; SD, standard deviation.

Note: Different uppercase letters indicate significant intrarow differences at *p* < 0.05.

In both groups, the symmetry of both muscles during MVC at the ICP was higher than that during chewing ( Table 2 ). When compared to those in the skeletal Class I group during chewing, skeletal Class III was shown to have a higher MM symmetry but a lower TA symmetry. Nonetheless, neither of them was statistically significant ( *p* > 0.05).

There was a significant difference in muscle synergy between patients in the skeletal Class I and III groups during MVC at the ICP ( *p* = 0.045) but not during chewing ( *p* = 0.978) ( Table 2 ). Less than 50% muscle efforts of both MM and TA were seen in skeletal Class I and III. Despite their nonsignificant differences ( *p* > 0.05), the effort of both muscles in skeletal Class III was higher than that in skeletal Class I ( Table 2 ).

## Discussion

This pilot study disclosed significantly lower ( *p* < 0.05) averaged muscle activity during MVC at the ICP of MM in skeletal Class III patients than in skeletal Class I patients. Several factors might contribute to this phenomenon. The volume and length ratio of the MM have been reported to be lower in skeletal Class III patients than in skeletal Class I patients, implying the lower muscle activity and force generation in the former. 31 Moreover, MM fiber orientations in skeletal Class III patients are less vertical to the mandibular plane than those in the skeletal Class I patients. This illustrated their lower mechanical advantage. 32 Occlusal contact number and surface area have been shown to affect muscle activity, 33 34 and tooth contact's sagittal position is important for muscle function during clenching. 33 35 In this study, the mean overjet of the skeletal Class III patients was negative and significantly different ( *p* = 0.001) from that of skeletal Class I patients. Consequently, their occlusal contact number and surface area, together with muscle activity, were significantly less than those of skeletal Class I patients. Despite their nonsignificance ( *p* > 0.05), the averaged TA activity during MVC at the ICP in skeletal Class III patients was lower than that in skeletal Class I patients. The explanation might be that MM dominates over TA during a high clenching level, 36 with a more noticeable activity than that of TA during MVC at the ICP.

MM activity in skeletal Class III patients during chewing was nonsignificantly lower than that of skeletal Class I patients in the present study. Similar results of lower MM activity during chewing in preorthognathic skeletal Class III patients have been documented. 37 Moreover, their MM activity during chewing in the postorthognathic period became as high as those in the skeletal Class I group. 37 A postsurgical increase in the occlusal contact areas might cause discrepancies during chewing in such patients. Food texture also affects muscle activity. 38 In the current study, the patients' MM and TA activity when they were chewing gum was approximately 30 to 44% of those during MVC at the ICP. Since the measurements of muscle activity during gum chewing were performed when the gum was softened, MM and TA showed less activity than those during MVC at the ICP.

An investigation into the muscle balance has suggested a more important role of occlusal stability than skeletal morphology. 39 However, skeletal and dental posterior crossbite have been documented to affect muscle balance. 9 26 27 In addition, patients with skeletal malocclusion show indifferent symmetry of the TA 26 and less symmetry of MM activity. 26 27 The indifferent TA activity might contribute to the patients' neuromuscular adaptation from their mild skeletal asymmetry. Since the patients possessed no asymmetry in the present study, nonsignificant intergroup differences ( *p* > 0.05) were detected in their MM and TA balance during MVC at the ICP and during chewing.

During MVC at the ICP, the averaged MM and TA synergy in patients in the skeletal Class III group was significantly lower than those in the skeletal Class I group ( *p* = 0.045), demonstrating their poorer muscle coordination. Averaged MM and TA synergy during chewing in the present study was nonsignificantly different, which coincided with those in a previous report. 26 The patients' neuromuscular adaptation might contribute to the nonsignificant difference in such synergy during chewing.

Masticatory muscles with a lower efficiency can cause a higher chance of muscle fatigue and disorder. 2 40 When compared to those with meso- and brachyfacial patterns, dolichofacial subjects presented with the significantly poorest masticatory performance but with the highest MM and TA effort. 2 Despite the nonsignificant differences ( *p* > 0.05) in the present study, muscle effort of MM and TA in the skeletal Class III patients was higher than those with skeletal Class I, indicating that such muscles in the former functioned more in similar activities. In the current study, a sagittal discrepancy of the skeletal Class III patients and their low muscle activity during MVC at the ICP might bring about a higher muscle effort.

Due to the coronavirus disease 2019 pandemic in Thailand throughout the current study's experimental period, almost 70% of the new orthodontic patients were excluded due to their reluctance to participate in the research. sEMG could be placed on the MM to detect its whole muscle activity. Nonetheless, some anatomical limitations caused us to detect only TA activity, leaving those of intermediate and posterior fibers undetected. If the muscle activity of the latter two fibers can be examined, a comprehension of the temporal muscle's accurate functions might be obtained. The results of which might be different from those in this pilot study and probably lead to an alteration in future researches' direction. Data from skeletal Class II patients were not shown, owing to their very small number ( *n* = 3), and are to be clarified. In addition, the muscle activity of patients with different skeletal relationships and food types (size and hardness) needs further investigation.

## Conclusion

Patients in the skeletal Class III group possessed lower EMG activity than those in the skeletal Class I group, but there was a significant difference ( *p* < 0.05) in only MM activity during MVC at the ICP. Within the limitations of this study, masticatory muscle performance in skeletal Class III patients was inferior to that in skeletal Class I patients; that is, lower averaged muscle activity, lower muscle synergy, and a greater tendency to muscle effort during jaw functions were observed.
